# Hair tourniquet syndrome of labia minora: case report

**DOI:** 10.11604/pamj.2022.43.68.22579

**Published:** 2022-10-10

**Authors:** Ahmed El Mouloua, Elouafi Elaouni Kamili, Karima Fouraiji, Mohamed Oulad Saiad

**Affiliations:** 1 General Pediatric Surgery, Cadi Ayyad University, Mother and Child Unit, Mohamed VI Teaching Hospital, Marrakesh, Morocco

**Keywords:** Hair tourniquet syndrome, labia minora, child, case report

## Abstract

Hair Tourniquet Syndrome (HTS) is defined as strangulation by a hair thread of the appendage of the human body, especially in the children population, it rarely occurs in external genitalia (clitoris, labia majora and minora). Herein, we aimed to present a rare occurrence of HTS around the labia minora in a 12-year-old child treated by excision of the cystic lesion with an uneventful follow-up. Special care should be given to every swollen appendage in children to avoid severe consequences such as amputation and disfigurement.

## Introduction

HTS is an emergent condition that requires immediate treatment. If the diagnosis is delayed, immediate complications such as necrosis of the involved area or amputation develop. It has been described for the first time in 1612, and the first case was published in Lancet in 1832 [1]. The HTS is a rare condition in which a human appendage is strangulated by a strand of hair. We report the tenth case in the literature of labia minora tourniquet syndrome in 12 years old girl.

## Patient and observation

**Patient information:** a 12-year-old girl with an unremarkable medical history presented to the pediatric emergency department with acute painful swelling of the right labia minora that appeared two days ago. No history of fever, vaginal discharge, or urinary tract symptoms was mentioned. Her family had no history of malignancy or other chronic medical illness.

**Figure 1:**
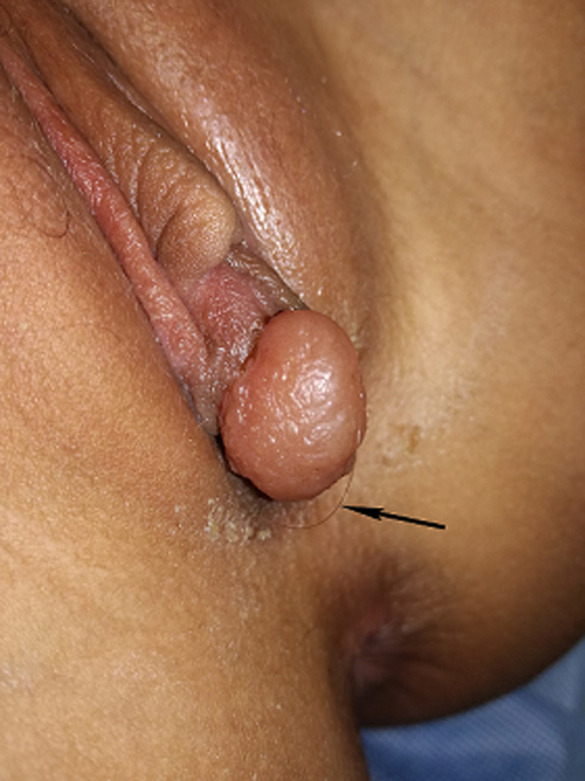
close view of cystic lesion of the right labia minora, with a hair tourniquet around it's distal-portion, this stalk of tissue is being suspended by a thread of hair (arrow)

**Clinical findings:** the physical exam the patient was in a good general state with normal vital signs. The vulvar examinations showed a 1.5 cm cystic lesion at the dependent portion of the right labia minora (Figure 1). It was painful indurate and tender with suspicion of a constructed hair band around it causing a tourniquet-like compression. The patient´s hymen was normal, without irregularity or scarring, and no other abnormalities were noted.

**Diagnostic assessment:** examination under a microscope showed a strand of hair wrapped around the labia minora.

**Figure 2:**
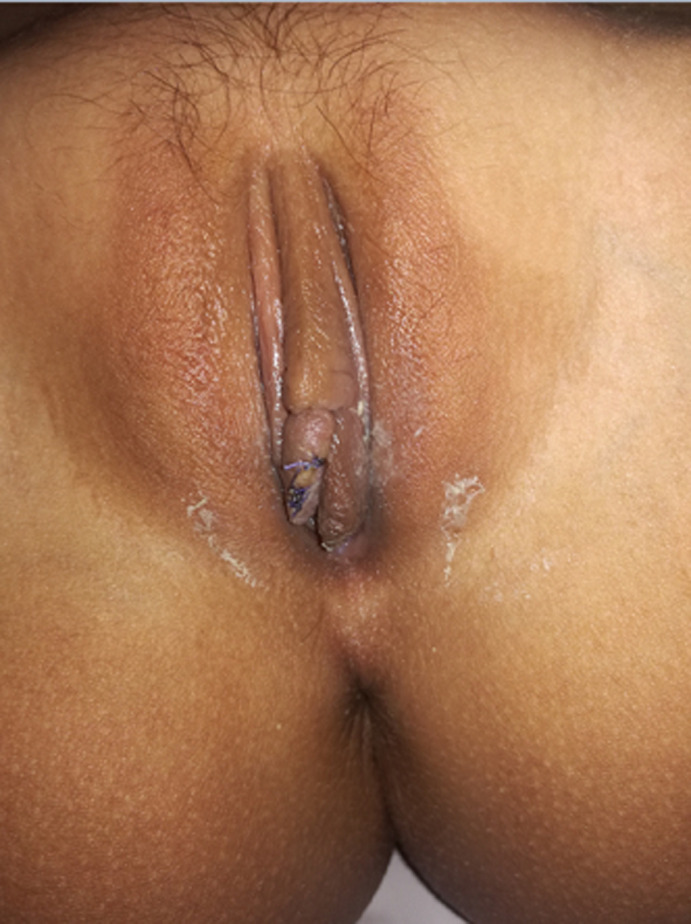
right labia minora after excision of the appendage

**Therapeutic interventions:** the patient was taken to the operating room, and an initial attempt of removing the wrapped hair was unsuccessful. The cystic lesion was excised entirely with no complication (Figure 2).

**Follow-up and outcome of interventions:** the postoperative course was uneventful, and the patient was released the next day, at a two-week follow-up, the patient was in a good health and pain-free.

**Patient perspective:** during treatment, the patient and her family were satisfied with the level of care provided to her.

**Informed consent:** written informed consent was obtained from the patient for participation in our study.

## Discussion

HTS is defined as strangulation by a hair thread of the appendage of the human body, especially in the children population. It´s occurring currently in fingers, toes, penises, and rarely in external genitalia (clitoris, labia majora and minora) [2,3]. This strangulation causes initially pain and oedema, which can lead secondly to tissue ischemia and amputation [4].

Only a few cases of labia minora tourniquet have been described, and this is the tenth case in the literature [5]. The incidence of the HTS is approximately 3.7% in previous reviews, with ages ranging from 10 to 14 years [5,6]. The strangulation of the appendages by the hair causes oedema and swelling because of the lymphatic and venous obstruction, which can lead secondly if not treated at the time to obstruction of arterial perfusion and may finally lead to amputation.

The diagnosis of tourniquet hair syndrome is based on the history and clinical exam which can find swollen appendages, oedema and tenderness. Magnification may be useful to identify the tourniquet. Every physician should be aware of this pathology and sexual abuse should be considered. Differential diagnosis may include infection, insect bites, allergic reactions and other [5]. The HTS is an emergent condition and adequate treatment should be instaured as early as possible after the diagnosis. Several techniques have been described in the literature such a mechanical release, excision of the appendage and recently a depilatory agent with thioglycolate was applied to release the tourniquet [7]. Nevertheless, the depilatory agent is contraindicated in female because of the proximity to the mucus membrane [5]. In our case, the mechanical release was not possible, and excision of the appendage was performed with no complication.

## Conclusion

Hair tourniquet syndrome involving the labia minora is extremely rare in children. Examination under a microscope makes the correct diagnosis and eliminates differential diagnoses. Every physician should be aware of this pathology and adequate treatment should be instaured as early as possible after the diagnosis.
